# Selections and Abstracts

**Published:** 1903-10

**Authors:** 


### SELECTIONS AND ABSTRACTS.

---

### THE NEGRO PROBLEM FROM THE PHYSICIAN'S POINT OF VIEW.*

*Mr. President and Members of the South Carolina State Medical Society:*

The magnanimous compliment implied through an invitation to deliver the Annual Address before this Association thrilled me with an amplified sense of importance and pride, and lest the declination of so great an honor should prove a never-failing cause for regret—to me—I immediately consented. Already a pensioner upon the bounties of South Carolina's hospitality, its medical skill, its health-inspiring air and sunshine—because of perennial exchanges for Southern comfort and strength of my acquisitions of Northern pains and weaknesses—it is meet that I confess here and now a gratitude inclusive of past privileges and present honors.

With the hope that I might bring to you a message equal to the full measure of the worthiness of this presence and occasion, I vainly sought a subject other than the one presented, viz: "The Negro Problem from the Physician's Point of View." No topic, however adequate, could gain advantage over my first election. Therefore it remains the "subject irresistible." If the message I shall attempt to convey betray weakness it is the more truly mine own; but if perchance some strength may appear or some nobleness unfold as if emanating from me, it is the reflection of the high human aspiration, brilliancy and warmth native to your people and skies, which guarantee to the Southern hearts and to this portion of God's world—perpetual summer.

The conviction has long possessed my mind that to the science of medicine must be entrusted the last analysis of the negro problem. The great questions that have exercised the minds of individuals and people revert for their ultimate solution to the physi-

*Read before South Carolina Medical Society, by W. T. English, A.M., M.D., Pittsburg, Pa.

cian. It was one of Pittsburg's most eminent physicians who, as one of the national arbitrators, suggested the first rational and acceptable terms of a cessation of hostilities between the North and the South. The truest histories and records of the past are those left by the physician. The most trustworthy arbiter and counsel of his day and generation, is the physician. He who would prognosticate with approximate exactness finds his best resources in that technical knowledge and experience appertaining to the study and practice of medicine. The widest views in this field as expressed by philosophers, philanthropists, moralists and jurists gain amplitude by visualizing from the physician's point of view. Nor is the cause for these special endowments of the physician far to seek. In addition to that perfectionment of vision secured by liberal education, included in medicine, there is a contact and a personal intimacy with human weakness and frailty, through medical practice, which contributes to judicial capacity. He reads the book of human nature while the people turn the leaves. The physician's response in the performance of his duties to human kind becomes habitual and self-acting, and the right is followed instinctively without a struggle, and not even the consciousness of the stress and strain of duty. This exercise of duty is coupled with discernment through which the seen expresses the unseen. Furthermore, the medical man has greatness of soul which dissolves personal distinctions. The high and the low, the rich and the poor, are equal subjects of his impartial benefactions. The true physician recognizes no limitations by creed or doxy and has no field of service less wide than humanity. To one thus endowed the moan of injustice, the cry of ignorance and shriek of pain are alike calls which inspire the activity of all the wisdom, courage and sympathy he possesses. These qualities in the physician of this twentieth century are supplemented by a charity which above all earthly goodness "thinketh no evil." There are times when the eminently good man may turn a deaf ear to human weakness and need, the generous man close his hand and the merciful man harden his heart; but the medical man continues to the end the monopolist of good offices. No human calling tends to minimize self and maximize beneficent offices towards others like to that of the medical profession. This is but a brief sketch of the medical man whom cyclopedias commemorate and whose name is graven upon the fleshy tablets of human hearts everywhere. It is the picture of the past, the reality of the present, and is exemplified by the myriad noble thoughts and countless self-sacrificing deeds of the physicians of South Carolina. Therefore I dare request that you follow me in the perusal of some thoughts upon "The Negro Problem from the Physician's Point of View."

The concern of the entire nation on one hand, and the physical, mental and moral welfare of millions of colored people on the other, await the secret analysis and lonely delving.

The objective and subjective cause and consequence may unfold under the scrutiny of the trained eye and earnest mentality of unbiased inquiry. Where human vision fails the omniscient eye of the microscope extends the field of the finite to the border lands of the infinite. The pains and penalties which human destiny ordained may be differentiated from those which are the direct sequelæ of violations of orthodox laws. There may be revelations which the subject, if animate, would fain conceal.

Discriminating the negro physical characteristics as they reach cognition through the medium of the sense of sight, we note his general appearance. Those who are the descendants of unadulterated Ethiopian stock and begotten beneath the southern sun are lacking in physical proportions in ninety-nine cases out of one hundred. A careful inspection reveals the body of the negro a mass of minor defects and imperfections from the crown of the head to the soles of the feet. The surface anatomy is full of blemishes which outnumber those of nearly every other race if careful comparisons be made. The negro skeleton admits of identification from that of other races because of peculiarities in growth which are general and special. To a general view the negro body presents a coarseness or rudeness and a variety in symmetry with other mathematical inaccuracies. The skull of the negro is demonstrably heavier, thicker and coarser in texture than that of the white man. Its surfaces are smoother, because the more complicated structures leave their impress upon the Caucasian skull. We may accept the evidence of the osteologist that the structure of the long bones of the negro, when examined microscopically show comparatively less minute capillary distribution. Moreover, there appears on the records of osteologists evidences of the difference in contour and structure of the negro skeleton corresponding with the age and environments in which the subject lived. The superior and inferior malar bones of the negro give to the facial outlines a peculiarity easily objectified. The feet and hands and relative length of extremities are racial marks which come out of the depths of centuries. As medical men we cannot fail to note an inferiority in his skeletal conformation and structure which intimates the primitive character of his physical framework. Moreover, the pathologic records are full of interesting details of the numerous diseases of the bony structures of the negro race. In some of the soft structures which overlie the skeletal framework there is apparent a grossness which would link him to animal ancestors. The dermal thickness of the black race is rarely observed in the paler races. This, together with puffiness beneath the eyes, broad and flattened nose, pouting lips, naso-labial folds, dull, apathetic face, abdominal prominence, exaggerated buttocks, inequality of the thighs and legs, the fibro-elastic increase around the heels and down the outer border of the foot, which is commonly lacking in the arching of the sole, and the picture of acromegalia, the suggestion of myxoedema, or some other trophoneurosis, present a possible pathologic solution. Enlarged and amplified laryngeal structure and cavity lengthen the vocal oscillations and deepen the voice, which is often wanting in proper delivery in consequence of the clumsy employment of a large and flabby tongue. If the conditions of the thyroid be investigated it will be found abnormal, ranging from hypertrophy to total obliteration in over fifty per cent. The general lymphatic conditions are subnormal with a tendency to take on hypertrophic growth from slight cause. In this respect differing markedly from those of the white race.

The diathetic and cachectic elements render the negro race vulnerable to Paget's disease, or osteitis-deformas, rickets, hip-joint disease, gigantism, exophthalmic goitre, thymic angiomatoses, and various associated psychoses—inclusive of syringomyelia.

The viscera and organic structures display a wantonness in contour, function and utility. The brain is lacking corticular area. The cerebral convolutions are not numerous, neither are the sulci deep. The gray matter of the negro brain is estimated to be at least 1,000 years behind that of the white man's brain in its evolutionary data.

Diseases of the structure of the brain have been on the increase among the negroes during the past generation, and epilepsy, cerebral softening, and other lesions appear in this portion of his history. This is a field for the histologist and neuro-psychologist. It awaits a larger investigation and greater results than have hitherto been obtained. The nervous system of the black race has not had the anatomical consideration needful to a fair physical comparison with other races, although the psychology of the negro has received a large measure of scientific study. If we depart from the mere anatomical consideration of neural growth, there are large and interesting revelations through the negro's general sense of feeling. The special senses are usually capable and they have a physical superiority over those of the white race when fairly averaged. Hearing, sight, touch, smell and taste average above those of some of the higher races.

The heart of the negro is estimated to be larger than that of the white man, and varies in weight, size and function, corresponding with his habits, to a larger degree than belongs to normal cardiac instability. The circulatory apparatus fulfils its functions with comparatively few delinquencies. The appearance of aneurism, arteriosclerosis, atheroma, varicose veins, angio-neurotic oedema, and other angio-neuroses, are not more common among the black than the white race.

The organs of respiration are apparently quite vulnerable, but average one ounce and a fraction larger than the white. Pneumonias and pleurisies are very common and fatal. Pulmonary tuberculosis threatens to settle the race problem by elimination.

The negro digestive apparatus is constructed for him, and by long adaptation has acquired a capacity to withstand privations alternating with irregular repletion, which seems to be the limit of human digestive tolerance.

In the genitourinary apparatus the widest possible human range is attained in the negro anatomy and physiology, as also in their associated psychologic influences. The external organs are extremely variable in size, and it is in the negro race that we find the miniatures and monstrosities.

Let us turn from these references to the period of beginning life, corresponding to the protoplasmic evidence of vitality, as expressed in the primal capacity for elasticity and contractility, and view, if you please, the mighty mystery which is the forum of every race—and permit the pre-natal panorama of cause and sequence to become mentally sentient with all possible completeness and celerity. The thousand details of embryology take many of us back nearer the beginning than the end of our earthly journey. How well you remember first the embryonic heart throb within the ovum which appealed to the sense of sight within the field of the microscope. Your consciousness admitted that virility had its first exercise and beginning in protoplasm; that sentiency became obvious with the nerve structures. You have continued the study until you may affirm that the compounding of ganglions in the encephalon is needful to volitiency of will.

Here, in this very ante-chamber of life, there are laws with which we, as medical men, have become conversant, causing arrested development and its sequalæ—monstrosities. In rudimentary organs with which these monstrosities are associated, the primal law of regression is established. The fœtus arrested in development at the lanugo becomes a human curio or hairy "dog-man"—whereas, had the arrest been postponed, a mental phenomena—a philosopher, may have resulted. Life in all its parts demands in the investigation a more complete analysis than this meeting will permit. This casual view can only be effective by prompting to a desire for comprehension of the human life and its complex, and a special application to the study of "The Negro Problem from the Physician's Point of View." Who will take up this field of investigation and its relation to the monstrosities so common in negro gestations?

After all the exigencies of an eventful gestation have been met, and the human being feels the realities of independent existence, the influences of heredity and environment assert themselves.

The negro has not had a large physical or mental realm from which to elaborate himself. He must have the same life history in protoplasm, in embryology, and all the other multitudinous steps leading to independent existence, which have been yours or mine.

The subject hitherto has been approached only from the anatomical side. We noted the crass anatomy and its construction, and have intimated some comparisons. The argument has taken, in swift review, the protoplasmic beginning of life and the strictly physical phenomena incident to pre-natal existence. The influence of that which succeeds, as far as it can be considered, must be included in general rather than specific detail.

All the phenomena of the negro life dependent upon material body, in a material world, and controlled by laws fitted to material things, are results of actions and reactions upon, and modified by, physical conditions.

Observe the advent of the negro into the world, under the Southern sun, which inclines to debilitate him. His progenitors of a race habituated to coercion to induce them to performance of duty, dependent upon the stimuli of accidental surroundings for impulse and incentive to services in their own behalf or for others. Truly his are the belongings of nothing more than the essentials of physical existence. We may add that the negro, thus emerging, has an exaggerated appetite and abnormal increase of feeling. These additions unrestrained by development of moral sentiment from within, or physiologic apprehension, due to somatic appreciation of functions, or other influences from extraneous source, and we have the negro legacy, at birth, fairly estimated. As growth proceeds, he continues as purely physical as human life may be. The stimulus of the mighty race in whose presence he lives and moves and has his being, accelerates rather than inhibits an innate tendency to sex appetite. It will not be forgotten that the negro morals were not regarded with reference to his sexual purity, and, indeed, it will be admitted that the development was ever toward activities needful of change. Furthermore, that laxness or wantonness remains as a part of his ancestral inheritance. At the present time the animal continues wherever he is.

Each recapitulation of the generation under like conditions must emphasize this sex-diathesis, adding the variations of impulse peculiar to those individuals contributing to the life.

Imagine an army of human beings thus feebly equipped by birth and circumstances of environment, suddenly deprived of restraints hitherto imposed by a higher race, and upon which it had been dependent for direction, but now forced to lean upon its own impotent resources. A race of human beings with instinct for self-preservation, coupled with a perverted sexdiathesis as the only incentive to continue in existence. The primal instincts of conjugal and maternal affection subjugated by a wide range of the subhuman. The paradoxical sexuality appears at this stage of human life whereby indulgences become ever more and more the incentives to over-feeling and passion, rather than toward quiescence.

There are creatures whose only purpose in life is to propagate their kind. Their birth, growth, life-mission, and death in hoary age, are compassed by the brief span of three score minutes. The psychology of these ephemeral specialists would be interesting "from the physician's point of view," and might suggest the moral conclusion that heaven has little time for life, with no higher incentive than self-gratification of the animal feeling with propagation of its kind. The measure of life, however, is not to be computed by its length of days.

> "That life is long which answers life's great end;
> In hoary youth Mathusalams may die."

Consciousness measures life with an accuracy which forces mathematical calculation from vital tests.

The ephemera may be possessed of a quickened consciousness through which he receives the same impression and measure of his life as we, and his three-score minutes may appear to him the equivalent of our human length of years—three score and ten.

The physical is the element upon which this earthly existence depends. It is the basis for all superstructure, mental and moral. But it must not be estimated as the life. Consciousness differentiates the vegetable from the animal existence. By and through consciousness, that which is animal in the race, is elaborated into that which is human. Moreover there is development consequent upon its slow but persistent influence which evolves the human element into perfectionment that has kinship with the divine.

In the negro races, the lower levels of consciousness are the more influential because of their senurial prerogatives. But there is little in human experience out of which to weave an empiric psychology or metaphysics of the sexual side of human existence. The fairest and most profound thinkers have assumed that sexual extremes belong to the age of awakening consciousness, or nascent intelligence, a stage of incipiency to moral and mental development.

It has been asked: "Has the negro arrived at the full status of man?" Let him who has attained that status, in all that it implies, press this question as the qualification for entrance into the field of human opportunity.

Full of imperfections and deviations from a model or standard which are in the main physiologic and not pathologic, he is a physical man. Nor is it quite hopeless if he have only the physical. There are those for whom we strive who have less than that.

It appears that the negro sex-diathesis is the most prominent and obvious factor in the physical predominance over the will. That the largest display of the sex-diathesis is concomitant with the smallest development of the psychic element may be accepted as a rule. There are numerous human exceptions. It is the observant and confidential physician who can estimate better than any other earthly being, how much remains on earth of the primitive animal sexuality which is scarcely concealed, and, on the other hand, can attest to the equivalent of martyrdom endured by men and women who are under the thrall of passions which make daily calls upon psychic and moral reserve. Every manifestation of unrestrained sexual lawlessness thus becomes possible in the midst of sexual self-control of the holiest and most sacred type. Sexual man is equipped as an animal. The influence of morality and intelligence dominate his conduct, and therefore the dominion is essentially psychic. In the negro the psychic differentiation is withheld, while the organic sex development continues. A sexual animal exists without the concomitants of a sexual man.

It is one of the laws of evolution that development may come by atrophy. Through arrest in development in organs once useful under primitive conditions, they become vestigial, fall out of use, disappear; or leave but homologous traces of their former existence. By this method of adaptation, it is possible for new organs to enter into the life and mediate the functions incident upon more complex relations. Nor is it the organic alone which disappears, but the psychical equipments of the past also becomes useless and are eliminated. Instincts are as important as corporeal structure for the welfare of the species under the present conditions.

In general, the changes, psychic and somatic, are gradual, and often tedious. Since the fishes in the Mammoth Cave began their life of total darkness, hundreds of generations have waned and the organs are not yet obliterated.

The physical origin is more easily traced backward by comparative anatomy than psychic or mental attributes by the comparative psychologist; but the research is worthy of every devotee to physical and psychic science.

The negro, by some demonstrations in the recent past, seems to be living over the instincts and habits of his animal sub-human ancestors, and in his extremities of moral delinquencies, corresponds with the point of arrest of development. Sense repletion is the earliest evidence of decadence, and finds its commonest expression in beastiality and gratification. The conditions of gratification are side by side with primitive instinct, coupled with inferior intelligence. The results appall us; but they are not mere accidents. They are the sequalæ to a diathesis, and not mere chance.

A perversion from which most races are exempt, prompts the negro's inclinations towards the white woman, whereas other races incline toward the females of their own. In comparison with the Indian, the diferentiation emphasises the negro sex-diathesis. The Indian gust was for blood, the negro for beauty. Some self-constituted philosophers have threatened to take the negro by cross cut over the centuries which have been necessary to the evolution of the white race, and place him in position for which he will not be qualified in a thousand years. This is a wrong which cannot be condoned. To extend to a race, through false teaching, an egotism which should only be acquired through gradual evolvement from rational humility, is criminality to that race.

To make the negro the equal of the white, a vast complex of interests and physical eliminations, inconceivably remote, must visit him. Psychic elimination has scarcely begun in the negro. Moveover, there is a lamentable but not quite hopeless inertia in the negro soul. The native opposition of inertia to vital force is not counteracted by activity. The lift must come through physical effort if at all. To touch the zero mark needs but inertia. High potential range is attainable only by painstaking effort. It must be impressed upon the negro that there is a life history, belonging to each, through which he must pass before he can reach the borderland of development. There is a phylum or tribal route elected by birth which he must overcome. Intensity of instinct feeling must be reduced. The negro race is an old one, and there must be vouchsafed a heritage of organic and psychic debris which will remain because of creative inequality to the task of counteracting the same.

The mighty efforts of nature are with the negro, and are in touch with all reasonable exercise of energy. But he must bide his time. As long as it has taken to subtract a superfluous rib, he must wait to be adapted to his plane of possibilities, and it were well that he should be impressed with the fact that his present conditions are far beyond the race of which he is an insignificant unit, and that if he would add one neural cell to the perfectionment of his racial evolution, he must drone no longer in the sense-benumbing sunshine.

In estimates of sex relation, sex-diathesis and attempts at analysis of the negro problem, there have been many secured from absurd points of view. The legal profession has posed in attitudes unwarrantably judicious and presumptuous. From the sublimated atmosphere of the sanctuary there have emanated some remarkable revelations of precocity seeking their own condemnation.

It is not because of a need for more numerous suggestions in the remedial measures that the subject is rehearsed before you here, but it is the physician's point of view which should be objectified at this juncture. It has been overtreated by those who are not qualified. Many able treatises have sought the light, that are worthy alike the cause and those expounding them, and show fairness from the point of view taken. That but few physicians have attempted to secure a view-point is because of their natural reticence prompted by the undesirable phases of the subject. We have patiently read some of the most incompatible prescriptions that could have been compounded of human words and symbols without one protest. There have been several most remarkable morsels of nonsense added to the literature of the subject during the past year, extending the therapeutics of the negro problem from an Eddyitic standpoint. The author sees, or perhaps only thinks he sees, the negro's situation contracted into a divine spark that has nothing to do but to glimmer, and in the chaos of word-painting which succeeds, gets the negro and his problem, the divine spark and God, into an inextricable confusion of relations with each other. This disciple of Christian science had never been near enough to the negro to give him anything but absent treatment. It may be remarked that the negro problem has received much absent treatment from others than Eddyites. Emphasis must be made upon the need of a nearer treatment, such as may be obtained through the physician's point of view.

With such an attitude of physical, mental and moral laxness before us, and with many opportunities within us, and the special qualifications for observation and study of this problem, it behooves us to reason from the physician's point of view that we may ultimately suggest the remedy. Treatment must not be ignored. The neural delinquencies due to faulty structure or lowered tone, may await kindly skill. The irritability of physical structure may respond to some therapeutic anodynes. Perhaps some anaphrodisiac of ample potency is within reach that will tame the bestial impulse. What has not medicine accomplished in its efforts for human relief?

It may become needful to call upon surgical aid for elimination. The honor of one Caucasian woman secured against the assaults of a sexual beast belonging to an alien race must awaken suggestions from the great medical heart of South Carolina, which will echo from the Atlantic to the Pacific. Moreover the study of each negro, as he comes within the range of suspicion, should become the duty of the physician. If the display of sex-element places him under suspicion, a system of investigation and observation by a committee with legal authority, should decide his fitness for liberty.

Education is always a step full of promise in the elimination of faults of mind. Its benign influence is inclusive and not exclusive. We look with hopefulness for its results. But that it be speedily so demands special antidotal qualities for sex-diathesis.

A great mathematician once made the statement that no erotic impulse could withstand the opposition of a mental solution of a problem in vulgar fractions. Deep study and abstract thought are logical counter-irritants to sensual impulses. They are immediate and enduring and are capable of individual application to the needs and adjustable as the dosage by the subject. They have not hitherto had a fair trial upon the negro over-feeling. They are natural in this sense at least. The capacity of the negro for abstractness in thought will determine the physiologic possibilities of this remedy. Education, as such, will never destroy the sex-diathesis.

Another panacea for the condition of the negro is suggested by the moralist. It is religious oversight and direction. It will suffice to call attention to the history, sacred and profane, attesting to the alliances of religion and sexuality. It is not my capacity to discuss the worthy influences of religion which reach out into this avenue of human need and racial good. But lest it might appear that this discussion would detract from the sexual instinct, I pause one moment in its defense. The sexual has been the element in the greatest achievements recorded in the history of the world. Races, nations, principalities, powers and their religions and people have been the offspring of this all-animating human instinct. All nature attests to its virile inspiration. To ignore or speak lightly of the sexual life is a hypocritical affectation, and intimates a virtue too sensitive of its exercise to be wholesome. Out of the depth of centuries comes the record of devotion to the sexual element through the god worship of Priapus. The maiden reaching the flower of her age was by public ceremony and ritual, adorned with a miniature emblem of the husky god in token of the aquisition of the loftiest womanly grace. This sex worship has been manifested in various ways during all the ages. Never has it received so many different interpretations and exercises as to-day. In conclusion permit me to remark that one effective means which would tend to lessen abuse and cure bestiality would be a more perfect comprehension of the dignity and possibilities of the sexual life. If such conditions obtained degraded life of lower races with their bestial spawn need not then appal us, nor feigned virtue dare keep death watch at the portals of our own race. The human nobleman would not barter his sexual birthright for aught else on earth. The true woman would be ever grateful and conscious that her body is a holy temple dedicated by God in which alone may continue the ever complicating warp and woof of evolution and life down with the ages yet to be, and thereby she becomes physically immortal.

---

### THE SURGICAL TREATMENT OF ABDOMINAL DISEASES.*

It must be conceded that the most rapid advances in surgery in late years has been in the treatment of diseases of the abdominal organs. This is due in part to the perfection of our surgical technique, and our practically perfect asepsis, and in a great part to our ability to diagnose more accurately the lesions and changes in these hidden organs. Our diagnostic ability has been much enhanced by pathological studies in the laboratory and on the living subject in the operating room.

In this short paper I shall touch upon only a few diseases which, in my humble opinion, can be best dealt with from the surgical standpoint. Let us take, for instance, that much discussed organ, the appendix vermiformis. Strange though it may seem, there are men in the profession to-day who do not believe in operating upon *any* case of appendicitis, maintaining that all will recover under medical treatment, or at least without surgical inter-

*Read by Louis Frank, M. D., Professor of Abdominal Surgery and Gynecology in Kentucky University, Medical Department; Surgeon to the City Hospital; Member of Surgical Staff of John N. Norton Memorial Infirmary, Louisville, Ky., by invitation before the Central Kentucky Medical Association.

vention. I am myself convinced that only rarely will a case of appendicitis die in the primary attack of the disease, provided it is properly treated. But just here is the rub. Our patients and their families will not aid us, nor will they comply with the rigid rules which must be laid down and followed out in managing the case properly. How many of your patients can you hold if you refuse to give relief by morphine, or some anodyne? How many of them are willing to be fed per rectum for a week, and to forego even drinking of water or some most bland nourishment taken by mouth? Very, very few. I am firmly convinced that no case of primary appendicitis ever completely recovers unless the organ is removed. Examinations made in numbers of cases have convinced me of this. The first attack subsides, but behind is left either a scar, a constriction which causes retention of fecal contents distal to it and a subsequent enterolith, or a small ulcer or an infected wall ready to again give rise to trouble upon the slightest provocation. Practically every case of perforative appendicitis, every case of appendicitis with enterolith, and these are quite frequent, have undergone one or often more previous attacks, each one increasing the dangers to the patient either with and most certainly without operation. There can be in the minds of those who honestly and thoroughly investigate this subject but one opinion, and that is that the disease demands the presence of the surgeon even if only in the role of consultant from the beginning, and further, that early and primary operation is by far he best plan of treatment, yielding the best results with least danger, quicker recovery, and saving more lives than any other method we have.

Should, perchance, as a result of surroundings or inability to procure a competent surgeon, it be impossible to operate, then absolute quiet in bed, withdrawal of all and every kind of food or drink by mouth with rectal feeding, and no purgation or opium, will be most satisfactory. Early diagnosis and early operation are, however, the slogan. The diagnosis is as a rule easy, and rarely in my experience has a mistake been encountered in overlooking this lesion or calling it some other disease. On the other hand, I have not infrequently seen cases diagnosed as appendicular disease in which the trouble was in some other organ. This is notably true where women have been affected, here inflammatory disease about the appendages, in one case an ovarian cyst, in another an ectopic gestation being the real trouble. As a rule these cases were operative, so that the gentlemen with whom they were seen made no mistake as to treatment, the indications being clearly surgical, and I was able to sustain them in their advice to their patients. In one case seen the physician in attendance had construed an attack due to gall stones as appendicitis. Operation for the former was followed by complete relief, no disease of the appendix being found. Movable kidney in woman and renal calculus in the male have also been the occasion of a call for operation for appendicitis.

In women, then, especially, we may mistake other diseases for appendicitis, and I am sure this is done far more frequently than the reverse, though the other diseases of the right abdominal region must always be considered if the patient is a female. In the male peritonitis means practically always appendicitis. The exceptions are infrequent, no matter where the pain may be located or referred. I was enabled recently to see this very forcibly demonstrated, and, I am sure, to impress it upon the mind of one of my medical friends.

I try to instill this into the minds of my students and believe that the time we spend in the study of this disease in our course is well spent, and we are fully repaid if we can get these few points fixed firmly in their minds:

1. That appendicitis is a dangerous disease.

2. That the symptoms upon which to base a diagnosis are pain in the belly, greatest tenderness over the base of the appendix, which is the McBurney point, no matter where the pain may be referred or located, and rigidity of muscles at this point.

3. Do not wait for tumor, as often no tumor ever forms, and oftener the patients are beyond surgical aid by the time tumor is present.

4. Do not be guided, or rather misguided, by pulse or temperature.

5. Peritonitis in the male means nearly always appendicitis.

6. The disease is surgical from the beginning.
7. The first twenty-four hours is the time for operation.
8. If operation is refused or impossible, starve the patient through the attack and operate in the interval before a recurrence.
9. Never, never, never give opium in any form.

*Gall Bladder Disease.*—If there is one surgical procedure which yields results which are entirely satisfactory to the patient and to the operator, it is that done for thè relief of gall stones. In speaking of gall bladder disease I shall not take up but one, cholelithiasis, and of the various locations of stone we will exclude all but those in the bladder. The diagnosis once made to the satisfaction of the attendant, there is, I contend, no treatment which relieves completely, quickly, and that, with practically no danger, and a loss from business and accustomed duties of not more than three weeks, like cholecystotomy and drainage. It is simple, quick, and if all stones are removed, one of the most gratifying operations in the entire domain of surgery. Medical treatment may relieve symptoms temporarily, but never permanently. I wish here to express my opinion most emphatically that neither olive oil nor any other medicament ever cured a case of gall stones or caused their passage. The passage of stones which have come away after possibly months of treatment, of pain and of suffering, is only coincidental, and would have occurred without any medicine. The passage of these stones is not effected by a dilatation of the ducts, but by ulceration or the formation of a fistula between the bladder and intestine. There is further, even should a stone be passed, no assurance that the bladder is free of concretions.

A bladder stone means an infected bladder. The treatment of necessity must be by drainage. This can only be accomplished by opening the viscus by a surgical operation.

An infected organ is a menace not alone to health, but to life. The sequellæ, such as perforation and peritonitis, hemorrhage, obstruction and cholemia or intestinal obstruction, or the frequent occurrence of cancer which follows impaction in the ducts, cannot and must not be gainsaid nor overlooked. They are not fanciful but real dangers.

In this disease we may, however, experience some trouble diagnosis. Many cases are doubtless now and will for some years be treated as cases of dyspepsia and indigestion. If you will investigate your cases of digestive disturbances carefully and thoroughly you will find numerous cases of gall bladder disease. Do not wait for jaundice to appear, for this is a symptom in only a small percentage of cases and occurs only as a result of inflammatory or calculus obstruction of the common or hepatic duct. Pain and especially tenderness on pressure at Robson's point, *i. e.*, midway between the ninth costal cartilage and umbilicus, is the most valuable sign. In conjunction with a history of long-continued dyspepsia or indigestion, especially shortly after eating, paroxysmal, radiating into back and right shoulder, with relief following vomiting, is almost pathognomonic. Oftentimes there is just the slightest tingeing of the sclera with bile.

The one reason why we do not see more cases of gall stones is simply because we do not look carefully for them, but are satisfied with treating these patients from year to year by dieting and digestants and those remedies used for a supposed functional indigestion. One point I would also mention and have you remember is that gall bladder colic may exist quite severe and no stone be present. Operation will reveal a distended bladder filled with bile or bile and mucus, no stone, no apparent obstruction, but drainage will relieve completely.

One case seen was the mother of a prominent newspaper man, who had suffered for years with indigestion and repeated attacks of paroxysmal pain in the gall bladder region. There was slight jaundice with vomiting during the acute attacks. At operation the bladder was found distended and enlarged until it reached below the umbilicus, a fistula was established, and, after a month's drainage permitted to close. The result has been a cure of all symptoms. No dyspepsia nor pain for over a year, and she is, I think, cured.

*Renal Calculus, etc.*—In kidney stones we have a condition which in its early stages may be entirely amenable to medical treatment. Renal stones may, however, rapidly increase in size; often they are large when symptoms first manifest themselves, and under such circumstances nothing short of removal by the knife can do any good. We should be alert when we hear complaints as to pain in the loin or in the abdomen for this condition. I have known patients to die from the effects of renal stone and their presence never suspected. They are insidious in formation. The symptoms are often obscure, not classical, and therefore they are often overlooked. I attach greatest importance to blood in the urine, ofttimes only in microscopic quantities, pain on pressure of the kidney between the hand in the loin, and the hand superimposed on the abdomen over the organ, and tenderness on pressure at a point midway between the auxilliary line and the umbilicus, and on a plane with the latter. The X-ray in the hands of some expert operators has yielded brilliant results, and, if diagnosis is uncertain, is worthy of a trial.

Should one be assured of the presence of a concretion nephrotomy is the operation of choice, and unless the kidney was hopelessly destroyed a nephrectomy should never be done. The operation is rapidly done and comparatively easy of performance through Morris' oblique incision, which I personally prefer, believing the organ can be more easily lifted out of the cavity for examination and incising, and more room given for manipulation than through the perpendicular incision down the back. The hemorrhage, while alarming if not properly controlled, is ordinarily of no moment. The poles of the kidney should be avoided by the incision, which goes through the organ to its pelvis, and should be made a bit posterior to its middle line and longitudinally. After the pelvis is opened the calices may be easily palpated and any stones removed. The exploratory incision into the kidney and digital palpation is far more certain and preferable to needling indiscriminately in all directions in the search for the stone, and the latter should not be done either in place of the incision or as the preliminary thereto. If there is no pus in the kidney it may be closed with catgut sutures passed deep into the parenchyma without drainage of the kidney itself, though for fear of leakage it is a good idea to drain the renal fossa for twenty-four or forty-eight hours.

*Movable Kidney.*—About fi.teen or twenty per cent. of all women 1 examine present movable kidneys, in many of these no symptoms being, however, referable to the mobility. Since observing these patients closely my opinion has undergone some change in regard to the treatment of these cases. Formerly such patients in whom this condition caused symptoms were treated by properly constructed corsets, and some were benefited, others still suffer, a few are comfortable with the corset, but possibly only one cured. Two of these cases, bad ones, I have recently advised to be operated on, for since noting and comparing my results after operation with the non-operated, I am forced to conclude that it is the thing to do. And it cures by an operation which in itself is free from danger and puts the patient in bed for about three weeks. This condition frequently follows labor with perineal tears.

A case operated on four months ago has been completely relieved. She had angulation of the ureter, a large kidney, and suffered much from urethral obstruction. Dangers present were those of cystic kidney, due to distention from pressure from retained urine. Pain agonizing. In proper cases nephropexy is certainly the one plan to be advised and even urged.

*Suppression and Bright's Disease* as treated by decapsulation I have had no opportunity to test. In the former, however, it is a life-saver in certain cases, and Edebohls has reported many brilliant and almost miraculous results following the operation in the latter.

Of the many other abdominal diseases, such as those of the female generative organs and the pancreas, we will not have the time to touch even in as hurried and brief manner as the foregoing, but I cannot conclude this subject without a few remarks in regard to typhoid and traumatic perforation of the intestines, bowel obstruction and cancer of the stomach.

In these latter conditions, as in most other conditions intra-abdominal, the important point is to make an early diagnosis and to follow up this by an early and complete operation.

*Typhoid and traumatic perforation* are to be treated in all instances by immediate surgical intervention. In traumatic perforation due, for instance, to a gunshot or stab wound, it should not be so much a question of waiting until the perforation is demonstrated absolutely by subsequent symptoms, but to take it for granted that these wounds are all of such character and that opening the abdomen is demanded. Only by following this rule can the greatest good be done and best results achieved. These injuries in civil life are of such a nature that they practically never recover without laparotomy, even though the intestine is not perforated.

The conditions surrounding the patients are such that there is no danger at all from an exploration *per se*, and we should not therefore base the treatment of wounds of this character upon the results achieved by non-surgical treatment in those wounded in the abdomen on the battle-field. In the latter the missile is of entirely different character, the surroundings and conditions under which the wounds are received are not conducive of the best operative results, while on the other hand the velocity and smallness of the projectile, in conjunction with the fact that the intestines are usually empty or nearly so, are great factors in getting the many recoveries following non-surgical treatment.

I believe that the time has now come when we should be prepared to treat perforations, the result of typhoid ulceration, by immediate celiotomy and closure. The brilliant results which have been achieved in this class of cases, which we look upon ordinarily as hopeless and in which recovery is almost miraculous, treated along the old lines, should convince us not only that this is the right and proper thing to do, but should also cause us to be constantly on guard and watchful for the earliest indications of this complication. I place more reliance upon sudden and sharp pain in the abdomen, especially the right lower quadrant, followed by symptoms of shock, though at times in the very slightest degree and rapidly succeeded by pain more or less localized, with rigidity of the abdominal muscles, as most valuable signs of this accident. Distention may occur very rapidly, and if liver dullness disappears and tympany takes its place, that is, if free gas in the abdomen is demonstrable, the diagnosis is almost certain. To wait until distension appears, however, or until symptoms of general peritonitis develop, will be ofttimes postponing interference too late. Certainly with general peritonitis, suppurative as this peritonitis usually is, there is not much chance of success in a patient who has been sick for two or three weeks with typhoid fever. The operation when done should be done then as early as possible after the diagnosis is made, should be performed rapidly, and, if a general anesthetic cannot be borne, be done under local anesthesia, wasting no time in useless or unnecessary pottering, and finally the abdomen should always be drained, and thoroughly so.

*Bowel Obstruction.*—The only point I desire to mention in the treatment of intestinal obstruction, whether due to band, volvulus or hernia, is that the time has come to cease manipulative efforts in the hope of relieving the condition. In this day of modern surgery to keep a man under an anesthetic for an hour or more attempting to reduce by taxis a strangulated hernia, and certainly to do this without chloroform, or to fool away time trying to relieve an obstruction by rectal injections, by distention with air or posture, is, in my opinion, criminal. The reason so many of these cases die may be summed up in two words—meddlesomeness and delay. Every case of intestinal obstruction, unless due to cancer or an inoperable tumor or an impacted foreign body, should recover from an immediate operation—that is, just as soon as a diagnosis is made, provided the diagnosis is made early in the case; and I may say, in passing, that there should be no difficulty in the diagnosis.

*Cancer of the Stomach.*—The results of Robson and Mayo in the magnificent work which they have been doing, and the experience of all surgeons in the treatment of malignant disease, is such that we feel we can safely recommend early surgical treatment in these cases; it is the only hope for cure. Careful attention must be given in the future to the symptomatology of gastric diseases. All symptoms referable to the stomach must be carefully investigated and we must perfect ourselves in our diagnostic methods and ability in reference to this organ. I think I may safely say that all doubtful cases should be explored, and if conditions are found amenable to radical surgery they should be relieved. Many times, though, radical means cannot be used, palliative measures may be carried out much to the relief and comfort of the patient and the prolongation of life.—*Louisville Journal of Medicine and Surgery.*

---

***NOTICE.— This Journal and the International Journal of Surgery one year for $1.00 cash, to new subscribers. No out-of-town checks accepted unless 10c. is added for cost of cashing. Strictly to new subscribers.***

### THE TREATMENT OF FRACTURE-DISLOCATIONS OF THE SPINE WITH INJURY TO THE SPINAL CORD.*

The subject of the treatment of fracture-dislocations of the spine associated with injury to the cord, must be of vital interest to any one who comes often in contact with these cases, for in the light of our present views, there is no case more distressing than a total transverse lesion of the cord.

I have selected this subject not with the hope that I might throw any new light upon the field, but that by reviewing the authorities I have at hand, the subject might be placed in a clearer manner before our eyes, and discussion excited upon a few points.

Before taking up the matter of treatment I wish to touch upon the subject of diagnosis, without going deeply into the matter of regional localization, for the necessity or advisibility of any active treatment must depend upon the nature of the injury inflicted upon the cord, and not upon the fracture of the bones themselves. The condition of the spinal cord is the essential point.

The points to be determined are these: Is there a total transverse lesion of the cord or is the interruption of conduction due to partial lesion only, the nature of which may admit of remedy by active interference?

Of the partial lesions we will have to consider contusion (embracing so-called concussion) and compression, due either to direct pressure from the dislocated fragments or to pressure from hemorrhage or inflammatory exudate.

The essential point of diagnosis is the determination of the total or partial lesion, and if but partial, then to determine as far as possible the nature of the partial lesion, and the possibility of remedy by interference.

That it is possible to distinguish between a total and a partial lesion a resume of the symptoms of each class will show.

The signs of a total transverse lesion of the cord are definite and positive. There is immediate and permanent flaccid paralysis of all muscles supplied by nerves given off below the lesion, with total anesthesia of all areas at the same level. There is absence of

*Read by W. W. Richardson, M.D., Bessemer, Ala., before the Jefferson County Medical Society, May 25th, 1903.

all kinds of irritation. There are no convulsions, no jerking of the muscles, no shooting pains. The parylized limbs are not stiff, but flaccid, the lower extremities for example being rotated outward from the weight of the feet. Only on the upper border of the paralysis remain contractions and radiating pains. Within the paralyzed area, however, all motion and sensation are absent. The bladder and rectum are likewise paralyzed and there exists retention or incontinentio urinæ et alvi. If this fails there is no total transverse lesion. The blood vessels share in the paralysis as shown by dilation of the subcutaneous veins and increased local temperature. Another vaso-motor symptom, which may or may not be present, is priapism depending upon the seat of the lesion and age of the subject.

One of the most important signs is the condition of the reflexes, especially of the deep reflexes. In a total transverse lesion of the cord, the patellar reflex is immediately, permanently and absolutely abolished, and this is an important factor in diagnosis. The superficial reflexes are as a rule absent at first, but may reappear later. The absence of the deep reflexes is one of the positive signs of a total lesion, but as will be shown later the fact of their absence does not necessarily imply a total lesion. Their presence, however, excludes the total lesion. The reflexes from the genital organs may be retained or even exaggerated, especially in young men, and we may have erection and ejaculation by touching the parts and by pressure on the bladder, testicles, perineum or thighs. Also the bladder and rectal reflexes may be retained in spite of bladder and rectal paralysis. The electrical reaction remains at first normal.

The flaccid paralysis, the anesthesia corresponding to the same level of the cord, the immediate and permanent absence of all deep reflexes, and the paralysis of bladder and rectum, are always constant in a total lesion, and the absence of any one of these signs disproves its existence.

The partial lesions, by good history and careful observation during several days are as a rule, to be distinguished from the total, chiefly by the retention of even a slight grade of motion, or stiffness of the muscles or by the persistence of sensation below the seat of injury. If there is the slightest retention of motion or sensation, if the deep reflexes are present, if there is the slightest grade of stiffness of the muscles, areas of hyperesthesia, or darting pains in the paralyzed limbs, there can be no total transverse lesion. True, in a partial lesion, temporarily all signs of conduction may be absent, but in a short time, usually in twenty-four hours, often in less time, signs of conduction will reappear. The deep reflexes may be absent at first, and, as I have seen in two cases, may be permanently absent, but as a rule they reappear and become slightly exaggerated. The paralysis may be total with retention or incontinence of bladder and rectum, the anesthesia may be complete—in fact all the signs may be those of total lesion, but in a short time some signs of transmission through the lesion appear, usually first a return of sensation or of shooting pains in the paralyzed areas, and we know that all communication is not cut off. This is of importance, if the signs are ever so light, for in a soft structure like the cord, if a portion could remain intact it is not probable that the remainder has received an injury from which recovery is impossible.

Especially characteristic of partial lesions is the greater involvement of the motor than of the sensory area. It frequently happens that with total motor paralysis, permanent or temporary below the lesion, the sensibility remains normal or only quantitatively changed. In every case in which the sensory paralysis is wanting or is incomplete, or does not correspond to the same segment of the cord as the motor, one can be certain that there is only a partial lesion. The presence of shooting pains and hyperesthesia in the paralyzed area are positive indications of only a partial lesion. It seems to be generally accepted that the patellar reflex reappears after a time in partial lesion, in fact it is stated as an absolute rule, but in a case under my observation some years ago, in which paralysis and anesthesia appeared first on the third day following the injury, and became total, the patellar reflex remained absent after motion and sensation had reappeared. A similar condition is noted in a case now under observation, though it may reappear later. I believe it is normally absent in some cases.

Retention of urine is never absent in total lesion, and though it may be and often is present in partial lesions it may be absent when there is total paralysis of the lower extremities as it may be present with no other paralysis except of the rectum; as in one of my cases in which a fall upon the sacrum produced retention of bladder and rectum with no other signs besides anesthesia of penis, scrotum, perineum and a small area over the sacrum.

Frequently the paralysis of the bladder in these cases will be found incomplete. There may be with the retention, completely retained sensibility of the bladder and rectum, in fact there may be hyperaesthesia with severe tenesmus. Priapism may be present, but is not persistent as in total lesion. In a vigorous young man the absence of priapism in a high lesion would lead one to hope the lesion was not total.

The prognosis of transverse lesion is bad. With an exception to be mentioned later, all authorities I have consulted state unequivocally that regeneration of the severed cord is impossible. On the other hand, in partial lesions the prognosis is in general favorable, for if part of the cord has escaped injury the remainder is probably not very seriously damaged, and though the paralysis may be severe and extensive, much of it is due to pressure from extravasated blood and lymph and disturbance of circulation with swelling of the axis cylinders. Another reason for favorable prognosis is that a permanent lesion is due to destruction of nerve cells, while a destruction of conducting fibers only is capable of restoration, if the homologous fibers of the other side are intact, as shown in hemilesions.

*Treatment.*—Great care should be exercised in the transport of these cases, as it is quite possible for a cord which has been but slightly damaged by the accident to be irreparably injured by unskillful transport. I have seen broken backs carried by the shoulders and heels, and the only chance the poor cord may have had, destroyed by such handling. A bed should be prepared with a firm, even mattress. No pillow should be placed under the head. The position should be dorsal in spite of any deformity, with pillows placed under the back to exert pressure where needed. With exception of a fracture in the cervical region all forms of extension or fixation by plaster of paris are apt to do more harm than good.

Posture with suitable support by pillows will do more to overcome deformity than extension, which will not be well borne.

The greatest care must be observed to prevent bed-sores, and probably the water mattress is the best means to this end. They usually appear, however, in spite of all care. The bete noir of these cases is the lack of control of the bladder and bowels. The latter should be kept confined as much as possible. That where there is retention or incontinence of urine the bladder will be infected sooner or later has been my experience, irrespectively of what care is taken to prevent it. Allowing the bladder to fill and overflow is advised by some, but is attended with the danger of injuring the mucous membrane from circulatory disturbances and rather favoring than retarding infection. Henle advises permanent drainage with Nelaton catheter. When the bladder becomes infected frequent irrigation must be used to prevent extension and pyelo-nephritis from which most of these patients die.

The cases of total transverse lesion of the cord are by all authorities, with the exception hereafter mentioned, regarded as wholly beyond any possibility of recovery. They may live for years in low lesions. One of my cases was alive six or seven years after the injury. Another I followed for three or four. It is in the cases of partial lesion that the question of operation comes to be considered. The question of immediate operation in these cases is still open for discussion, though I believe the majority of our best authorities are not in its favor, and it seems to me with good reason. We cannot always differentiate, immediately after the injury, between the partial and total lesions, as severe contusion or pressure from extravasated blood may cause complete interruption of conduction along the cord, and only the lapse of several days will enable us to distinguish between them. Or if we know at once the lesion is partial we cannot say whether it is due to causes which will disappear of themselves, as slight contusion or extravasation of blood and lymph, or whether if due to pressure by encroaching bone it will be possible to remove it better by operation than by posture. Only when there is reason to believe that the lesion is due to pressure from depressed laminae, does immediate operation seem advisable, when the case is analogous to depressed skull fracture.

Here by removing the depressed spiculae the pressure may be at once relieved. These cases are, however, rare, and due to direct violence, while the vast majority of broken back are fracture-dislocation of the bodies from bending of the whole spine.

With the late or intermediate operation the case is different, for here at times much good may be done after any improvement which has taken place has ceased or signs of retrogression have appeared. Occasionally pressure may thus be removed and circulatory disturbances corrected with benefit. But even here it is difficult to say how much credit is due to the operation. I know that many brilliant results have been reported from early operation, but the skeptic will ask how much of the result is owing to the operation; and would the result not have been the same by expectant treatment, for many seemingly severe lesions recover rapidly. It is a difficult matter to judge. I have had no personal experience with the late operation. Several cases of partial lesion which have been under my observation, have shown such decided and steady improvement that I have let well enough alone, and they passed from observation still improving. I have operated immediately upon several cases, but all have proven to be total lesions, and should have been left alone, for though none died from the operation and one lived for years afterward, of course, no benefit was obtained from the operation. I have two cases now in hospital which illustrate nicely the two classes of partial and total lesion and a review of their histories may prove interesting.

CASE 1.—Thomas Holmwood, male, age 28, color, white, single, carpenter, admitted April 13, 1903, to Readers Hospital.

*Diagnosis:* Fracture dislocation of the spine. Partial lesion of cord at about fourth lumbar segment.

*History:* Patient was caught under a heavy timber frame, but exact mechanism of injury unobtainable.

Examination showed posterior curvature along entire dorsal region without any marked angular deformity. He states that this condition has always been present. The spine of the twelfth dorsal seems slightly more prominent than the others and there seems to be a slight depression of the first lumbar.

There is a total flaccid paralysis of the lower extremities with paralysis of bladder and rectum. The deep reflexes are absent but cremaster and abdominal are present.

There is loss of all sensation in both legs anteriorly to the knees, and upon right thigh nearly to the groin. Posteriorly the anesthesia extends upon the right side to above the sacrum, but upon the left side the posterior surface of the thigh retains sensation. The sacral region, buttocks, perineum, scrotum and penis are anesthetic.

*Treatment:* Dorsal posture on a hard mattress with pillow under dorsal curvature. An attempt was made to avoid catheterization by allowing the bladder to overflow, but at the end of twenty-four hours the distended bladder caused pain and the catheter was used. The urine contained considerable blood, albumen, and hyaline and blood casts. For three days the amount of urine was very small, and catheterization was necessary but once in twenty-four hours. On the second day his condition was precarious. The abdomen was tympanitic, and the stomach refused to retain anything. The pulse was rapid and weak. The vomiting became continuous with regurgitation of a dark greenish fluid containing flecks of black resembling disorgized blood. After washing out the stomach and giving a colonic flushing his condition improved, and by the fourth day his general condition was much better. Examination showed that the anesthesia was diminishing, and he complained of shooting pains in the paralyzed limbs. On the thirteenth day examination showed that the anesthesia was much diminished and there was present very slight contraction of the left sartorius. The bladder became infected in spite of rigid precautions in catheterizing. The urine began to overflow or could be expressed by pressure over the pubes. At present his general condition is good, but a small bed sore is present over the sacrum. The bowels are incontinent, but do not move very frequently. The sphincter, at first relaxed, is now closed. The anesthesia has diminished markedly, being present now only over the feet and outer posterior surfaces of the legs. The thighs are sensitive. The sacral anesthesia is diminished, and scrotum and penis show only diminished sensibility. The contraction of the sartorius is now marked, but there is no other return of motion. The deep reflexes are still absent.

CASE 2.—Will Bird, male, age 30, colored, occupation miner, admitted April 20, 1903, to Fossil Hospital.

*Diagnosis:* Compound fracture of the dorsal vertebræ with total transverse lesions of the cord at about the twelfth dorsal segment.

*History:* Patient was struck on the back by falling sand-rock. Examination showed flaccid paralysis with total anesthesia below umbilicus. The anesthesia extended to the umbilicus anteriorly and to about the level of the second lumbar spine posteriorily. All reflexes, superficial and deep, were absent. The penis was somewhat swollen, but not erect. There was a small wound in the median line of the back at about the junction of the dorsal and lumbar region from which escaped what appeared like cord tissue. As the fracture was already compounded it seemed best to operate.

*Operation:* Ether. Incision in the median line. The muscles of the back were found badly lacerated. A fracture-dislocation was found of the eleventh and twelfth dorsal vertebræ. The upper segment of the spine was dislocated forward, the articular processes of the lower vertebræ coming prominently into view. There was complete separation of all articulation, the fragments moving readily upon each other. The finger could be passed anteriorly along the bodies which were badly fractured. With forceps and chisel the laminae were removed from three vertebræ and the canal opened. The dura, in shreds, could be seen within the canal, but the cord was totally disorganic over an extent of about two inches. Above and below, the crushed cord could be seen oozing out of the canal. To attempt suture was ridiculous. As an assistant said: "You might as well try to suture thick cream." Without attempting more, the wound was closed with silk-worm gut, leaving a small gauze drain.

On the ninth day he was sent to Readers Hospital. His general condition was good. The anesthesia and paralysis had not changed. The bowels and bladder were incontinent. The urine was ammoniacal and full of pus. Now on the thirty-fifth day his general condition is still good although the temperature runs high, probably from infection of kidneys. There has been no change in the paralysis nor anesthesia.

The operation wound healed by primary union without a particle of infection.

Case one illustrates the salient points of a partial lesion, for though the paraphlegia was complete and all deep reflexes persistently absent, with retention of urine and faeces, the anesthesia was characteristic of partial lesion, in that it did not correspond to the same segment of the cord as the paralysis, and was not equal on both sides. The anesthesia corresponds to a lesion of about the third sacral segment while the paralysis is that due to a lesion of the third lumbar. The steady decrease of the anesthetic area, the return of motion in the sartorious, and the shooting in the paralyzed area, proved that there is still communication through the lesions. If this improvement ceases I should advise operation, but now I am inclined to let well enough alone.

Case two illustrates a typical case of total transverse lesion at about the level of the twelfth dorsal segment. The only excuse for operating upon this case was the existence of a wound communicating with the fracture. Infection could be best prevented by enlarging the wound for thorough cleansing. Moreover I was anxious to prove again to myself the impossibility of suturing the cord in these cases. That it required renewed proof was owing to an article by Drs. Stewart and Harte, of Philadelphia, which appeared in the Philadelphia *Medical Journal* of June 7, 1902.

They report a case of gunshot wound of the spine at the seventh dorsal vertebræ with total transverse lesion of the cord and separation of 3-4 of an inch. After resection of the seventh and eighth laminae the cord was sutured with catgut. All the signs of total transverse lesion were present. These signs gradually disappeared, the deep reflexes returned, the anesthesia diminished and by the sixteenth month the patient could flex and extend the toes, legs and thighs, and stand alone with support. I received a letter from these gentlemen in April of this year, saying that the improvement had continued.

This case is certainly interesting. Our greatest authorities may be mistaken regarding the possibility of regeneration of the cord. However, in the cases under consideration, I do not believe suture is possible, owing to the extensive destruction of the cord, which is usual.

The subject of greatest interest is the question of advisability of immediate operation in the cases of partial lesion, and it is this point which I hope will excite discussion.—*Ala. Med. Journal.*

---

### SOME OBSERVATIONS ON THE CAUSES AND TREATMENT OF ACNE.*

Acne, though exceedingly common, the most common of all skin affections, is one to which the profession at large, knowing that life is in no way endangered and that it is chiefly a malady of adolescence, to disappear with full adulthood, gives but little heed. Yet in my experience it is a disease which may persist for years and, while usually in no way detrimental to the general health, is not infrequently the expression of persistent ill-health and so disfiguring as to be most repulsive to the afflicted.

It is not necessary to define the disease, or to go into a description of the symptoms; or to relate the different varieties named according to the clinical appearance of predominating characteristics, as they are well known to you all, except to say that in general acne is an inflammatory disease, usually chronic, of the sebaceous glands, especially the glands of the face, the shoulders, the sternum and the trunk; characterized by papules, tubercles and pustules, often single but usually commingled; most commonly met with between the ages of thirteen and thirty years, between adolescence and adulthood. I except from this the so-called acne artificialis, due to the use of the bromides and the iodides and to the local use of such irritating medicine as tar.

The course of the disease is also familiar to you. Nor is the diagnosis difficult, except that I would warn you that rarely an acute case of acne (due generally to the ingestion of medicine) has been mistaken for smallpox; and I would guard you against the

---

*Read by Andrew P. Biddle, Detroit, before the regular quarterly meeting of the Monroe County Medical, Societv, July 16, 1903

not infrequent mistake of confounding acne, especially as found in large numbers upon the back, with syphilis. Acne rosacea is so closely identified with acne as to cause, effect and treatment as to require but little necessity of differential diagnosis. In the latter there are simply acne lesions in association with the dilated blood-vessels of the nose and neighboring tissues.

I would, however, invite your attention to the varied causes and to the remedies most usually found to be at least of some value. And in this I shall not make the attempt to go into the subject in a systematic text-book manner, but shall confine myself more to personal experience and opinion.

With the advent of puberty the hairs all over the body, both the strong and the lanugo, take on increased activity; with this activity the function of the sebaceous glands, which function is to lubricate the hair and the skin, is accentuated; and with these changes come functional and inflammatory derangements. The sebum secreted by the gland becomes inspissated, due to the thickening (keratosis) of the corneous layer; the duct becomes patulous, sometimes obstructed, and the comedo is formed. The black head is supposed to be due to the pigment derived from the secretions, or to some chemical changes due to exposure to air and light, or to alterations in the process of secretion, the result of some constitutional derangement influencing nutrition. The mass acts as a foreign body and by its presence creates a soil favorable to the growth of microorganisms; inflammation follows and the acne lesion is formed. Again errors of digestion cause chemical and irritating changes within the gland itself and create a favorable soil upon which microorganisms flourish. Often, it is supposed, the presence of a mere lanugo hair is a sufficient irritant.

The inflammation begins either in or around the gland, may extend to the surrounding tissues, and often dermic abscesses are formed. The contents are usually sebaceous matter and tissue debris bathed in a sero-purulent fluid. If severe, this is followed by destruction of the glands, to result in disfiguring and permanent scars.

In an article entitled "The Etiology of Acne Vulgaris" in the *Journal of Cutaneous Diseases* for March, 1903, Gilchrist (Johns

Hopkins University) states that he found definite bacilli, which he names the "bacillus acnes" present in all smears taken from 240 typical cases of acne from 86 patients. He assigns this as the primary cause of acne and would lay strong stress on the local cause of the disease, attributing the constitutional disturbances so frequently met with in the chronic cases to sepsis from the continued absorption of toxin produced by the innumerable number of bacilli present in the acne lesion.

Except in cases in which there are large pustular formations, in which condition there is undoubtedly added to the "bacillus acnes" a factor of local infection, I strongly believe in the predisposing constitutional cause; but when we see the disease in use of great physical strength, in training athletes, the difficulty of finding a predisposing cause is apparent. On the other hand, in the anæmic and the tubercular, the constipated, the dyspeptic and the rheumatic, in the neurasthenic, the over-fed and the over-indulgent, we can at least find a plausible explanation.

That errors of diet, over-indulgence in indigestible foods, as beer, cake, candy, rich foods, tea and coffee, is accountable for some cases can be readily proven by the rapid return of the trouble when the patient has committed the indiscretion.

The circulatory weakness, gastric and intestinal disorders, menstrual irregularities and pelvic diseases, strumuous and gouty diathesis, and many of the obscure reflexes, sexual over-indulgence and sexual abstemiousness, local irritations, as exposure to the heat of the cooking stove and fire furnaces, are accountable for the acne is exemplified by the improvement which takes place when it is possible to remedy the underlying cause. How these conditions cause the state of acne is not fully understood; but it is believed that "in a more or less lowered vital action of the skin the cells fail to carry through their process of fatty disintegration to a perfect result."

The acne is a rebellious disease, still under a well-directed treatment very gratifying results can be obtained. Here, as in the case of all diseases, the cure depends in a great measure first upon our ability to recognize the predisposing cause, and secondly upon our ability to remove it either in whole or in part; and I know of no disease which calls forth more distinctly the trained powers of observation, the knowledge and the skill of the general practitioner.

In the majority of cases the treatment must be both constitutional and local. The first step is to trace either directly or by elimination the source of the disease. There is little in the condition of the lesion to guide us, except that the flat, or slightly elevated, reddened acne lesion is most often associated with the anæmic and is very difficult of cure; the acne limited to or most predominant upon the chin is concomitant with some irregularity of menstruation and is usually aggravated with the approach of the menstrual epoch; the acne found in the crease of the nose, extending slightly to the cheek, is dependent upon some gastro-intestinal disturbance, most frequently constipation; and the large pustular, indurated acne is frequently mostly due to local infection, and is the most amenable to treatment. So in a general way we must carefully regulate the diet, advising the patient to abstain absolutely from all food known to be indigestible, such as pork, veal, pastries, gravies, cheese, the excessive use of coffee and tea, candies, sweets, highly-flavored ice-cream, etc., etc. In short, the treatment must be directed to the probable cause as suggested by your experience as a practitioner after a careful examination of the patient.

As a routine treatment, if there is any tendency to constipation, I give the ext. cascara sagrada, ½ gr.; ext. rhubarb, 1 gr., and ext. nux vomica, 1 1-10 gr. in capsule, three times a day. If necessary this may be varied with the many other laxatives, as the aloin, strychnine and belladonna pill, calomel, gray powder in connection with the various alkaline waters. To the dyspeptic may be given as indicated the bitter tonics, the alkalies, the acids, pepsin, pancreatin, the saline laxatives and the alkaline diuretics; to the chlorotic and to the anæmic, iron, arsenic, strychnine and manganese; to the tubercular and the debilitated, cod liver oil, iron, strychnine, etc. I have never seen any benefit from the use of the calx sulphurata, though it was at one time extensively used in all forms of acne. In selected cases some benefit may be derived from the use of ergot and ichthyol and of sulphur.

In many cases, however, the routine treatment will not suffice, and a deeper cause must be sought. That the acne is frequently reflex there can be no doubt, and so, often the treatment must be directed to the intestinal tract, to the irritable pile and fissure, to the uterine discharge, to the long foreskin, to the irritable bladder, to the sluggish bowels, to the displaced uterus, to the pus tube, to the functionally and organically deranged nervous system.

I consider local treatment to be of the utmost importance, though I do not agree with those who advocate it as the only treatment. In my mind there can be no doubt that the removal of the inspissated sebaceous plug removes an offending body; and, as in any other surgical case pus would be immediately evacuated, so here as a routine treatment I advise the physician, after rendering the skin aseptic, to open up freely each acne lesion, to evacuate the contents, be they simply the sebaceous plug or the debris among the pus. This process must be repeated every day or two until the face is free from acne lesions and the treatment must not be left to the patient, but be done by the physician himself. I do this by incising each lesion with a sharp knife and removing the contents by the use of the extractor. Under no circumstances should the lesions be squeezed between the fingers.

It has been found that in all cases of disease involving the sebaceous glands the local application of sulphur is beneficial and so it is my habit to apply to the face every night, if it does not cause too much irritation, the following carefully prepared lotion:

| | |
|---|---|
| Freshly precipitated sulphur | 5 parts. |
| Freshly precipitated zinc sulphide | 3 parts. |
| Hyposulphate of potassium | 6 parts. |
| Sulphate of potassium | 6 parts. |
| Rose water | 80 parts. |

Especially is this of advantage where the face is oily and in large, pustular, indurated acne.

The primary pathological condition in the comedo and acne is a keratosis of the corneous layer; so that frequently it is well to produce a mild grade of inflammation by the use of the tincture of green soap, applied to the face every night, medicated if desired with 10 to 20 grains of resorcin to the ounce. Again, in an obstinate sluggish case massage, preferably induced by cupping, may be employed, followed by remedial applications.

I am not in favor of the steaming of the face; but I do believe that the tonic effects of the daily cold bath is decidedly beneficial; especially is this true of the acne of the back. In this latter case the bath should be followed by brisk rubbing of the back with a coarse towel and the application of the stronger remedies.

For years Dr. Geo. H. Fox, of New York, has advocated the scraping of the skin with the dull edge curet, evacuating the contents of the glands forcibly, following the treatment with the application of a soothing ointment of boric acid, calamine, etc. While this treatment will undoubtedly hasten the removal of the contents of the glands, it is in my opinion too harsh for general use; patients object to it. Again, glands have been opened and the electrical needle has been applied to the individual lesion; carbolic acid has been employed in the same way.

Of late years ichthyol has come into use in the treatment of acne in the form usually of an ointment, 30 to 90 grains to the ounce of cold cream or some other mild base; especially in the pustular form, often combined with the tincture of green soap, if more energetic action is needed.

And so many remedies might be brought to your notice. Their number simply indicates the obstinacy of these cases. The principle is the same; the remedies are antiseptic and more or less astringent; experience alone must be the judge as to their character, whether best applied in the form of an ointment or a lotion, whether stimulating or soothing. I would, however, urgently warn you against the employment for any length of time of any ointment to the upper lip or chin or to the lower part of the face of any young woman; its employment encourages the growth of superfluous hair, especially in one inclined to the growth of hair and who has already suffered from a long continued irritation from the disease itself, which alone is often sufficient to cause the unfortunate hypertrichosis.

At a recent meeting of the American Dermatological Association a prominent member stated that he had practically abandoned all other methods of external treatment than the use of the X-rays and has had no reason to regret the results obtained. The use of the X-rays in acne is based upon their power of causing atrophy of the sebaceous glands, destroying bacteria in the skin, and controlling the formation of pus. My experience has been favorable as far as the immediate result of the treatment is concerned; but sufficient time has not yet elapsed to determine the permanency of the cure. It is now well-known that it is not necessary to produce a dermatitis to secure results and I would caution against the use of any but a weak light. I usually give exposures every second or third day, distance of the tube 15 to 20 c.m., and have never caused anything more than a slight pigmentation, never except in one case the mildest reaction.

While most of the cases clear up, if they do not disappear, with adult life, many of them persist for years, especially the indurated type and more particularly as shown on the back, where are found the large dermic abscesses with unsightly scars. To meet with success in cases of acne the treatment must be persistent, extending over weeks and even months, much attention must be given to the detail not only of the external but of the internal treatment: the patient's daily life and habits must be thoroughly regulated. But when successful, and in the majority of cases we do meet with success, the results are most pleasant to the patient and to the physician.—*Journal of Michigan State Medical Society.*

---

### A NEW CLINICAL ENTITY (OSLER'S DISEASE).

In the August number of the *American Journal of the Medical Sciences* appears in full the original article by William Osler, M.D., Professor of Medicine, Johns Hopkins University, descriptive of a definite clinical entity new to medical science, characterized by "Chronic Cyanosis, with Polycythæmia and Enlarged Spleen." This disease, as described by Osler in this paper and read before the Association of American Physicians, has created interest in this new symptom-complex which many of his friends have designated as Osler's disease in honor of this painstaking, differential scientific inquiry into this group of symptoms. This study is based on cases coming under his own observation and from a number reported in the literature of medicine. After reporting the cases in full Osler gives an exhaustive analysis of the conditions found, viz., chronic cyanosis polycythæmia, moderate enlargement of the spleen with the presence of a trace of albumin in some cases, and pigmentation of the skin in several cases. He describes these features in detail, as follows:

*Cyanosis.*—"Naturally this attracts most attention and has been the feature which has led to further investigation. As is usual in all forms of cyanosis, it is most marked about the face and hands . . . the skin of the entire body was of a dusky blue. When first seen the suffusion of the conjunctiva and the prominence of the eyes adds a startling appearance to the patient. The cyanosis is more intense in cold weather, and is aggravated by any existing bronchial catarrh. On bright, clear days, with but little moisture in the air, it may lessen. The period over which the cyanosis has been noticed varies from ten years to three or four years. While constant it may vary greatly in intensity . . . There is no respiratory distress with the cyanosis. While the skin looks full and tense and the hands bloated, yet marked dilatation of the large superficial veins is not noted. On close examination of the skin, many fine, dilated venules are seen.

*Blood.*—The viscidity is greatly increased. All observers have remarked not only upon the unusually dark, but upon the thick and sticky character of the blood drop. An extraordinary polycythæmia is a special feature of the affection. The maximum blood count was 12,000,000 per cmm. (Cabot's case). In eight of the cases the count was 9,000,000 per cmm., and in the ninth 8,250,000 per cmm. There have been no measurements of the red blood corpuscles. The statement is made that in the polycythæmia of congenital heart disease the red blood corpuscles are smaller than in that of high altitudes. The percentage of hemoglobin has been high, ranging in one case to 165. Usually the range has been 120 to 150.

The specific gravity of the blood in one case was 1,080; in another 1,067 to 1,083. In eight of the cases the leucocyte count ranged from 4,000 to 20,000. As a rule in the majority of the cases it has been below 10,000 per cmm.

*Spleen.*—In seven of the nine cases the spleen was enlarged.

*Urine.*—In seven of the cases a trace of albumen was noticed, with hyaline, sometimes granular casts. The sp. gravity was low.

*Symptoms.*—The symptoms have been very varied. Most of the patients complained of headache, weakness and prostration. Attacks of nausea and vomiting were reported in some cases. Cough and shortness of breath were each present in one case. No fever in any case. The pulse was of high tension and the vessels sclerotic. There was no edema of the skin. The torpor, mental and physical; the sensation of fulness in the head, with headache, vertigo and in some cases nausea and vomiting, remind us of the symptoms to which mountain climbers and æronauts are subject.

*Remarks.*—Chronic cyanosis, a common enough feature in clinical work, is met with:

1. In organic disease of the heart, particularly in congenital malformation, in chronic myocardial and tricuspid lesions in children and adults, and in cases of adherent pericardium.

2. In certain diseases of the lungs, particularly emphysema, and in long standing pulmonary tuberculosis of the fibroid type. Practically there are only two conditions in which patients walk into the hospital or the consulting-rooms with extreme cyanosis, congenital heart disease and emphysema.

3. In methemoglobinemia of chronic poisoning with coal tar products, as antipyrin and acetanalid, etc. In this condition too, the patient may startle one by the marked cyanotic appearance.

There are a good many people whose normal condition is one of great fulness of the blood-vessels of the skin, so that in cold weather there may be marked cyanosis of the ears and of the face. . . . Cyanosis, local or general, indicates one fact—diminished oxygenation of the blood corpuscles. In the deepest cyanosis of the ear or of the finger tip the blood count may not be above 5,000,000 per cmm. . . . Occasionally most extraordinarily cyanosis occurs in adherent pericardium.

*Polycythæmia.*—There are two classes of polyglobulism—*relative*, in which the condition is due to a diminution in the quantity of the plasma of the blood, and *true*, in which there is an actual increase in the number of blood corpuscles. Much work has been done of late years on this subject. Relative polycythæmia is very common. It may be caused by a deficient amount of fluids ingested, which possibly may be the cause of polycythæmia of the newborn, more frequently it is caused by loss of liquids, either by (a) sweat; (b) diarrhea (by far the most common); (c) increased diuresis. (d) In another group of cases there is a loss of liquids by secretion or transudation, as in narrowing of the pylorus with dilatation of the stomach, and in the constant loss of liquids from the blood in recurring ascites. It is interesting to note that in some of these cases the polycythæmia is of a high grade and may persist for months, or for even years. It is not necessarily associated with cyanosis, as is in cases of dilated stomach and in diarrhea. There is also a toxic polycythæmia described in poisoning by phosphorus and carbon monoxide, which, too, is probably relative. The polycythæmia of vasomotor disturbances, after the cold bath and exercise, also comes in this class.

*True Polycythæmia.*—It is met with where there is a difficulty in proper aeration of the blood, as in high altitudes, or in heart disease, congenital and otherwise; and also in the obscure cases of this form here under consideration. . . It is by no means easy to offer a satisfactory explanation of the polycythæmia with cyanosis here under consideration. It does not seem possible to connect it in any way with the moderate grades of enlargement of the spleen, and yet there are one or two observations in the literature which are of great interest in this connection.

With our imperfect knowledge of the physiology of polycythæmia it would be premature to discuss at any length the pathology of this remarkable group of cases. We need:

1. A study of all forms of chronic cyanosis with polycythæmia, particularly those associated with heart disease and emphysema. (It is to be noted that the cases here reported have the highest blood count on record, much higher than the average in congenital heart disease or in dwellers at great altitudes.)

2. A more accurate study of the blood in this class of cases—the volume, the viscosity, the state of the plasma and the serum, the amount of hemoglobin, the specific gravity, and the diameter of the corpuscles. As increased viscosity of the blood, with resulting difficulty of flow, seems the most plausible explanation of cyanosis, it is especially important to test the viscosity by accurate physical methods and to determine the relation of the number of corpuscles to the viscosity of the blood.

3. The relation of the splenomegaly to the cyanosis and polyglobulism should be carefully observed. It may not be anything more than the effect of the chronic passive congestion."

Future investigation will determine whether we have here in reality a new disease. The clinical picture is certainly very distinctive; the symptoms, however, are somewhat indefinite, and the pathology quite obscure.

This clinical entity described by Osler so fully should keep clinicians on the alert to observe in detail cases presenting this symptom—complex. We would be pleased to receive reports for publication.—*Medical Fortnightly.*

---

### SHALL THE NEGRO PRACTICE MEDICINE?

BY W. T. ENGLISH, A.M., M.D., PITTSBURG, PA.

This question has been answered in the negative by the anthropologist, who proclaims that the negro race is disqualified by nature from entering into the higher vocations. By comparing the convolutions and sulci in the brain of the African with those in the white man's, the anatomist and physiologist estimate the negro to be at least one thousand years behind. Biologists and histologists establish the general fact that the germinative cells alone can transmit to descendants their influences and that hybridism tends to bring races upon equable planes, but deny these advantages to the mulatto. From this mongrel race is withheld the capacity for continuing viable through the number of generations requisite to evolve those attributes belonging to the higher races, and which no civilization can produce or destroy. The physical basis upon which all attainment depends for human kind, fails before maturing time.

Accepting these unquestionable deductions of scientists, we cease to search for traditional gleams of light in the the negro physician's past, nor do we longer question why his present medical efforts touch the zero mark; and, knowing that the shortcomings of his race are not amenable to correction, we confidently predict for his medical pretentions a hopeless future. Lack of definite purpose among negro students of medicine cause nearly fifty per cent. to abandon the study. Of those who reach graduation, four out of ten relapse into other callings, while only one in three continuing in the profession arrives at what might be called reasonable success. This retirement of the negro from medical aspirations might be accorded to him as worthy if it were the sequence to an appreciation of unfitness, but this fact is not in evidence.

The negro disposition to retrograde from vantage ground is characteristic. Emancipated from slavery recently in Hayti and elevated to higher possibilities they are already back to voodoo worship and cannibalism. Many special conditions favor this retrograde from medical practice.

The negro memory has the possibilities of acquiring encyclopedic knowledge, but in medicine these arsenals of learning become antedated sooner than in any other profession, and it is the exception for authorities to remain a decade unchallenged upon the medical man's book shelves. Incessant investigations and frequent discoveries yield to advancing medicine an ever-widening area with inconstancy of outline. The negro physician is disturbed by all this, and each new thought finds in his limited mentality an awaiting prejudice. In his efforts at diagnosis and prescribing, whether in emergencies or with deliberation, the negro physician betrays his incapacity for individual or mental self-dependence. In medical practice, as elsewhere, he is artificial, formal and unreflective. His prescriptions are copies of those of his white teachers, and his technical enunciations recall the echolalia of the feeble minded. When medicine moves forward, and his encyclopedic knowledge is antedated, he is without resource and falls behind or quits the profession.

The negro is disqualified from entering the profession of medicine by ungovernable impulses, which preclude the acquisition of quiet fortitude and deedful skill, so prominent in the white medical exemplar. The negro emotions are as unrestrained and dangerous to-day as they were before civilization touched him. Without system, divested of sympathy, and minus principle, the negro medical man dispenses his services. His social tendencies are undirected, indiscriminate and excessive, and by promiscuous exercise of these he departs from necessary concentration of mind and professional stability of purpose. It will be admitted that the social physician per se is seldom scientific and rarely trustworthy. It is the practice of the noblest of medical men to become more or less gregarious and "of the herd," seeking and finding mental and social affinities amid the writings and counsels, as well as direct companionship of those of his kind. Moreover the lazy habits of body and general sedentary disposition of which the negro can not or will not be divested are directly antithetic to the constant demands for the employment of physical energy made in the active exercises belonging to a medical life.

In view of these facts alone, without referring to the numerous other detail that might be called in evidence, it becomes the proper course to discourage negro medical ambitions. Teachers who direct their negro pupils into the medical path are guilty of a many-sided wrong. No training can fit for a medical life one whom nature disqualifies. It imposes an unnecessary and grievous burden to thus force upon any one a competition which from the very condition of things means defeat. Furthermore it has been demonstrated that only the best of human kind is truly eligible to the office of physician. He should proceed from among the survivals of the fittest and be in every respect the fittest of those who survive. Natural qualifications should be supplemented by ample training, so that act, thought and being shall bear the impress of a noble inheritance and experience gathered from myriads of ancestral generations. To one so equipped there comes a gentle but irresistible call to minister to physical ills like to that which bids men to minister in the temples of Almighty God. If any earthly calling be divine, medicine assuredly is.

Regardless of these considerations there are those who imagine that anything which wears a human countenance is eligible to the highest places and capable of exercising the noblest function. It is lamentable superficiality of judgment which assumes that the negro requires only civilization and encyclopedic knowledge of the medical curriculum that he may become the white physician's confrére. Rather direct him into the inelastic environment of the legal profession, where authorities once accepted abide unquestioned for generations and wherein the negro's capacities for imitation may avail in mimic oratory. Better far to direct the negro ambition toward the function of the ministry, where his parrot-like methods need not always mar the rendition of the "old, old story," and where encyclopedic knowledge endures always.

The right of a negro to become a man is undisputed. This denial of the higher planes is not a question of the race prejudice, but a law of nature—that races are unequal. Armstrong, Frizell and Booker T. Washington have expressed similar views, and they are better qualified to teach the negroes than leaders of political organization.

Give the negro the privileges of the ballot from property reasons, educational reasons or hereditarily. Teach him to be self-sustaining and self-respecting. Uplift him by every availing effort; but for unnumbered generations yet to be, the answer of nature and experience to the question, "Shall the negro practice medicine?" must be emphatically, "NO."

---

### NEWSPAPER MEDICINE.

The following excerpts are taken from a single daily paper, and serve to account in a measure for the prevalent erroneous popular ideas regarding medical matters:

CHICAGO MAN'S HEART MENDED.—An operation was made on the heart of M. P., who was stabbed in a fight, to save the man's life. At Mercy Hospital, where he was taken, physicians took out his heart and sewed it up. Then oxygen was administered continously, and it was said that he had a possible chance of recovery. The wound almost cut the man's heart in two.

DR. GARTNER OF VIENNA has just patented an instrument which records the pulse of a patient under the influence of an anesthetic. The instrument is fastened to the forearm and a graduated disc records the increase or retardation of the pulse. The experiments in the hospital of Vienna succeeded marvelously. It is hoped by means of it to prevent death during operations.

SURGERY FOR CONSUMPTION.—L. G. of Hampton, N. H., underwent a rare surgical operation here on Saturday at the Stamford Hospital, as a last resort for the cure of pulmonary tuberculosis. An examination revealed a large cavity in the apex of the right lung and an abscess in the upper part of the middle lobe. The physician in charge of the case was satisfied that the only chance the patient had was an operation that would establish drainage through the chest wall. On Saturday, accordingly, he resected five inches of the second rib, four and one-half of the third and four inches of the fourth. The pleura being bound down by adhesions was brought into the wound and stitched there, both cavities were located and the pleura was opened with a needle. The cavities were thoroughly cleaned and drained with a rubber tissue drainage, and the wound was closed with the exception of space sufficient for a drainage. The chest wall was strapped and dressed with a dry antiseptic dressing. A complete recovery is hoped for.

The optimism of the final statements in each of these instances is not calculated to instill correct physiological ideas into the popular mind.—*Ex.*

---

NO EXPENSE has been spared by the M. J. Breitenbach Co. in having the Chart accurate.

The original sketches were in water-colors by one of the leading Bacteriologists in this country.

Being reproduced in the minutest details by the best skilled artists in lithography.

There are sixty separate examinations represented, requiring the services of four skilled artists to make the original drawings from sketches.

Time consumed, twelve weeks each; or labor for one man forty-eight weeks.

The labor of proving same required the time of three provers five weeks each, or the total time of one man fifteen weeks.

Eighty lithographic stones were used for the drawings and proving original designs.

The time for transferring and printing of all colors was sixty-one days.

The Chart passed through the printing press sixteen times.

Colors used:—Black, yellow, red, dark blue, purple, buff, light blue, medium blue, medium purple, light purple, light pink, medium pink, green, brown, gray.

